# Borderline grades in high stakes clinical examinations: resolving examiner uncertainty

**DOI:** 10.1186/s12909-018-1382-0

**Published:** 2018-11-20

**Authors:** Boaz Shulruf, Barbara-Ann Adelstein, Arvin Damodaran, Peter Harris, Sean Kennedy, Anthony O’Sullivan, Silas Taylor

**Affiliations:** Faculty of Medicine, University of New South Wales, Sydney, Australia

## Abstract

**Background:**

Objective Structured Clinical Exams are used to increase reliability and validity, yet they only achieve a modest level of reliability. This low reliability is due in part to examiner variance which is greater than the variance of students. This variance often represents indecisiveness at the cut score with apparent confusion over terms such as “borderline pass”. It is amplified by a well reported failure to fail.

**Methods:**

A borderline grade (meaning performance is neither a clear pass nor a clear fail) was introduced in a high stakes undergraduate medical clinical skills exam to replace a borderline pass grade (which was historically resolved as 50%) in a 4 point scale (distinction, pass, borderline, fail). Each Borderline grade was then resolved into a Pass or Fail grade by a formula referencing the difficulty of the station and the performance in the same domain by the student in other stations. Raw pass or fail grades were unaltered. Mean scores and 95%CI were calculated per station and per domain for the unmodified and the modified scores/grades (results are presented on error bars). To estimate the defensibility of these modifications, similar analysis took place for the P and the F grades which resulted from the modification of the B grades.

**Results:**

Of 14,634 observations 4.69% were Borderline. Application of the formula did not impact the mean scores in each domain but the failure rate for the exam increased from 0.7 to 4.1%. Examiners and students expressed satisfaction with the Borderline grade, resolution formula and outcomes. Mean scores (by stations and by domains respectively) of students whose B grades were modified to P were significantly higher than their counterparts whose B grades were modified to F.

**Conclusions:**

This study provides a feasible and defensible resolution to situations where the examinee’s performance is neither a clear pass nor a clear fail, demonstrating the application of the resolution of borderline formula in a high stakes exam. It does not create a new performance standard but utilises real data to make judgements about these small number of candidates. This is perceived as a fair approach to Pass/Fail decisions.

## Background

Ensuring competence in clinical skills is central to medical and health professions education. The Objective Structured Clinical Examination (OSCE) was introduced to increase the reliability and validity of clinical skills assessment in medical education [1]. Since being introduced, the OSCE has become established as one of the leading assessment tools in medical schools and across many health professions education programs [2–7]. Previous studies provide extensive evidence supporting the reliability and validity of the OSCE [3, 8–14]. To improve reliability examiners are commonly provided with a predetermined checklist to use when marking an examinee’s performance using categories from Fail to Distinction and at times numeric marks are attached to these categories [15]. Among the possible categories is a Borderline grade which describes a level of performance that is neither clear pass nor clear fail [14, 16, 17].

Definitions of the Borderline grade vary [18–20], for example ‘Students who possess just enough knowledge to reject F-responses’ [20] or ‘a minimally competent graduate is one who meets the standard by the smallest possible margin’ [21]. That perhaps explains why Cizek described a borderline proficiency as ‘an abstraction in terms of performance’ [22]. The intangible nature of the borderline grade poses a challenge to assessors, since different individuals may have different understandings regarding what constitutes a borderline grade. By definition, clear pass and clear fail grade decisions mean that there is no ambiguity and therefore examiners are more likely to agree with each other. Nonetheless, even in these circumstances, the literature suggests that the impact of examiners on the assessment outcome is critical. For example, examiners reported that they felt less confident when awarding a fail grade than when giving a pass grade [23]. Biases, such as gender and culture, have been found to have affected examiners’ judgements [24], as has examiners’ familiarity with the examinees [25]. A recent study on an OSCE used in an Exercise Physiology program found that the examiners accounted for 24.1% of the variance in technical skills scores, whereas students accounted only for 4.9% of the variance [26]. A comprehensive meta-analysis estimated that OSCEs achieve an overall low reliability (<.60) and suggested that an OSCE ‘*does not guarantee reliable scores and accurate decisions about medical student*s’ [27]. This substantial evidence suggests that examiners’ biases are unavoidable when OSCEs are employed. Although not explicit in the literature, the biases discussed are more likely to affect students performing at the borderline level than when their performance is a clear pass or clear fail.

To date, the literature addresses the borderline grade issue by suggesting methods for defining cut-scores for the entire OSCE examination or for specific stations. Among these are the Angoff method the Borderline Groups Method, the Borderline Regression Method and the Contrasting Groups Method [28–37].

The AMEE guide no. 49 ‘How to measure the quality of the OSCE: A review of metrics’ favours the Borderline Regression Method (BRM) [38, 39] since it “*uses all the assessment interactions between assessors and candidates, and these interactions are ‘real*” [2] and is “*objectively based on predetermined criteria, using a large number of assessors and generates a wide range of metrics*” [2].

Guided by the principles suggested by G Pell, R Fuller, M Homer and T Roberts [2], a new method (The Objective Borderline Method, henceforth: OBM) for addressing challenges raised by borderline grades was introduced [40–43]. The OBM utilises the institutional predetermined criteria for clear pass and clear fail and uses all assessment interactions between assessors and candidates [2] to determine whether a borderline grade should be reclassified as pass or fail, which is argued to improve the reliability of the OSCE. This study reports the application of the OBM principles, when the OBM2 [42] was applied to a high stakes OSCE undertaken at the end of the second year of the Medicine program at UNSW Medicine, UNSW Sydney, Australia.

### Context

The UNSW Medicine program is a six-year undergraduate entry program awarding two degrees, namely Bachelor of Medical Sciences (BMed) and Doctor of Medicine (MD) [44]. This modular program consists of three phases, each of two years. Students undertake barrier examinations at the end of each phase.

Year 2 students sit a clinical examination at the end of Phase 1. This focuses on three domains: generic communication skills; clinical communication skills (medical history-taking); and physical examination skills [45]. Previous to the borderline method being introduced (OBM2, described in detail in the next section), examiners were able to offer one of four categorical grades: Fail (F); Borderline Pass (P-); Clear Pass (P); and Exceeded Expectations/ Distinction (P+), and numeric scores were generated from these grades as follows: (F) = 3; (P-) = 5; (P) = 7; (P+) = 9 (out of 10). When student performance was uniformly outstanding, that is they received only P+ grades, examiners were at liberty to upgrade the numeric score from 9 to 10 for details see: [46].

The motivation for seeking an improved method for making defensible Pass/Fail decisions for the borderline grades arose from concerns expressed by course and program leaders that clinical examiners were overly lenient and failed to fail poorly performing students, rather tending to award P- rather than F grades. It was noted that examiners’ free text comments often indicated unsatisfactory performances, while the grade awarded was a P- (borderline pass), observations which echoed concerns reported in the literature [47, 48]. In addition, concerns were expressed regarding the nature and meaning of the P- grade. The P- was described as a ‘Borderline Pass’, yet some examiners perceived it as a ‘pass with conditions’. Under this previous marking method (henceforth, ‘traditional method’) accumulated criteria results of two P-‘s and no fails was graded as an overall pass, but a student with three or more P-‘s failed the station. This had a logical flaw since the P- (or Borderline Pass grade) was neither numerically nor descriptively defined as a Fail. Finally, the Phase 1 OSCE generally had a failure rate of less than 1%, significantly lower than other barrier examinations in the Medicine program.

The introduction of the OBM2 [42, 43] to the literature provided an opportunity for the program directors to improve the pass/fail decision making for the OSCE’s in the Medicine program and it was conducted across all clinical examinations in the UNSW Medicine program in 2016. Implementation of OBM2 focused on the abovementioned concerns. The Borderline Pass (P-) grade was replaced with a ‘Borderline’ grade, which indicates (as per the examiner instructions) that the examiner is unable to conclude whether the student performance for a particular assessment criterion (item) was a clear pass or a clear fail. Using of the Borderline grade permitted examiners to award an undetermined pass/fail assessment where appropriate, and when insufficient evidence was available to make a clear decision, prevented examiners being forced to do so. The use of the undetermined ‘Borderline’ grade (B) thus aimed to achieve two distinct goals: mitigate examiner’s marking bias [25, 47], and reducing examiner anxiety in difficult cases, which at times might encourage examiners to give students the “benefit of the doubt” and award them with an unjustifiable ‘pass’.

Implementation of the OBM2 included a revision of assessment guides and alterations to examiner training, which occurred throughout the 2016 academic year and the OSCE occurred in November 2016. Four relevant Faculty governance committees, each including student representatives, independently discussed and approved the implementation of the OBM2. A contingency plan was prepared should the implementation of the OBM2 fail. OBM2 implementation replaced all the examiner awarded B grades with either an F or P as determined by the OBM2 algorithm.

The current study focuses on the implementation of OBM2 [42] in this clinical skills examination (OSCE) undertaken at the end of Phase 1. The next section (‘*The Objective Borderline Method*’) describes the OBM2 in detail.

### The objective borderline methods (OBM and OBM2)

#### The OBM

The OBM [40] is a method that yields an index from two independent proportions of responses to examination items when the possible responses are: clear pass and above (P); clear fail (F); and borderline (B), an undetermined grade. The two relevant proportions are: (1) the proportion of P grades among all the non-F grades; and (2) the proportion of B grades among all the non-P grades.

If the number of P grades is p; the number of F grades is f; and the number of B grades is b then:

The proportion of the B grades among all the non-P grades is: Pr(B) = b/(f + b).

The proportion of the P grades among all the non-F grades is: Pr(P) = p/(b + p).

The OBM index is the multiplication of these: OBM index = Pr(P) × Pr(B) = [p/(b + p)] × [b/(f + b)].

Thus the OBM index presents: the difficulty of not getting an F grade (i.e. getting a B grade) given a P mark is not achievable; and the difficulty of getting a P grade given all grades are above clear fail (>F grade). Multiplication of proportions is an acceptable practice for yielding indices derived from observations. [49] . Importantly, although Pr(P) and Pr(B) are related, they remain independent since a particular *proportion* of P grades among the P and the B grades *cannot* determine the *proportion* of the B grades among the B and the F grades (and vice versa). The OBM does not apply when there are no B grades, since no decision is required. The OBM applies when examination marks are on a continuous scale yet uncertainty exists regarding the cut-score separating pass from fail. In order to apply the OBM there is a need to determine the minimum score for clear pass and the maximum score for clear fail, whereas the scores that are neither clear pass nor clear fail are defined as borderline. Since the OBM is a multiplication of two proportions each of which is a sub-group within a group (i.e Pr(B) = b/(f + b); Pr(P) = p/(b + p)), the OBM index is always ≤1. Upon introduction the OBM was used to determine the proportion of borderline grades that should be re-classified to Pass; and 1-OBM index determined the proportion of borderline grades that should be reclassified to Fail [40]. Using this classification a cut-score could be estimated (the lowest borderline grade that was reclassified to Pass). B Shulruf, R Turner, P Poole and T Wilkinson [40] demonstrated that the cut-scores generated by the OBM were highly correlated when compared with cut-scores generated by other methods. However, known standard setting methods, including the OBM, still left the question of determining individual student borderline results unresolved.

#### The OBM2

Logically, for each assessment criterion one must either pass or fail but never both, since not passing means failing, and not failing means passing. Thus a borderline mark or grade (used here interchangeably) means that the examiner could not make a clear decision, most probably due to insufficient information provided to them during the examination. The OBM2 was thus introduced as a *pass/fail decision making* process to reclassify the indecisive borderline grades to the most likely decisive grade, either pass of fail. The OBM2 is not a tool for generating a cut-score but is a tool for making defensible decisions when there is uncertainty.

The OBM generates two indices: the ‘Difficulty’ of an item, when all responses to a single item given by all students are considered, and an index of student ‘Ability’, when considering all responses to all items within a construct given by a single student. The OBM2 uses these two OBM indices to make pass/fail decisions for B grades and works by these two OBM indices being calculated for each B grade. If Ability < Difficulty the B grade is reclassified as an F grade, otherwise if Ability ≥ Difficulty then the B grade is reclassified as P grade.

It is important to understand that although inspired by Item Response Theory (IRT), the OBM2 is by no means a form of IRT, nor is it an alternative to IRT models. The OBM2 is used only in relation to a particular type of examination consisting of three types of grades (Fail, Borderline, Pass and above) and its only purpose is facilitating the pass/fail decisions when borderline grades are awarded. Similarity to IRT is evident in that item difficulty and student ability are measured on the same scale, and this is where the OBM2 and IRT are most comparable. The OBM2 is applicable only when items underlie a single construct, in the case of our study, each of the three domains: generic communication skills; clinical communication skills (medical history-taking); and physical examination skills [45]. Previous studies demonstrated that, to yield a high level of accuracy, items need to be loaded on a single factor and yield at least a moderately acceptable level of reliability (Cronbach’s alpha>.60). [42, 50].

Table 1 demonstrates how the OBM2 is applied. This example is taken from responses to assessment criteria related to physical examination in an OSCE conducted at one of UNSW’s clinical examination sites. The OSCE consists of six stations, in which each had 12 assessment criteria related to the three domains [45] and there are 24 examinees. For each item one can be awarded an F, B, P or P+ grade which were converted to a numeric score F (=3), B (=5), P (=7) or P+ (=9) for analysis. This produces a “raw” score. OBM indices (Ability and Difficulty) were calculated for each item and each student when applicable (if no B grade was obtained, no OBM index was calculated). Then for each B grade a comparison between Ability and Difficulty was made as described above. The arrows on the right hand-side of the 5’s (=borderline) indicate whether the 5 is modified to 7 (↑) or to 3 (↓).The grades in this demonstration yield good internal consistency (Cronbach’s alpha = .749). Readers may scrutinise the table to see how the OBM2 works across students and items. This table is readily constructible using Excel™ and readers may test it using their own data.

**Table 1:** A demonstration of how the OBM2 is calculated

Items Student No.	1	2	3	4	5	6	7	8	9	10	11	12	F	B	P	Ability: b/(b + f)*p/(p + b)
1	5↓	5↑	7	7	9	9	9	7	7	7	7	7	0	2	10	0.833
2	9	9	7	7	9	9	7	9	9	9	9	9	0	0	12	
3	7	7	7	7	7	7	7	3	5↓	5↓	7	7	1	2	9	0.545
4	5↓	5↓	7	7	7	7	7	3	7	7	3	7	2	2	8	0.400
5	7	7	7	7	7	7	7	7	9	9	7	7	0	0	12	
6	9	5↑	7	7	7	9	7	7	7	7	7	7	0	1	11	0.917
7	7	9	7	7	9	9	7	7	9	9	7	7	0	0	12	
8	7	7	7	7	7	7	7	7	7	9	7	7	0	0	12	
9	7	7	7	7	7	7	7	9	9	9	7	7	0	0	12	
10	7	7	7	5↓	5↓	7	7	7	7	7	5↑	7	0	3	9	0.750
11	7	7	7	7	7	7	7	7	5↓	5↓	5↑	7	0	3	9	0.750
12	7	9	7	7	9	7	7	7	9	7	7	7	0	0	12	
13	7	7	5↓	5↓	7	9	7	7	7	7	7	7	0	2	10	0.833
14	7	7	7	7	7	7	7	7	7	7	7	9	0	0	12	
15	7	9	7	5↓	7	7	7	3	5↓	7	7	7	1	2	9	0.545
16	7	5↑	7	7	9	9	7	7	7	7	7	7	0	1	11	0.917
17	7	7	9	7	7	7	7	7	7	7	7	7	0	0	12	
18	7	7	7	7	7	7	7	7	5↑	5↓	7	7	0	2	10	0.833
19	7	7	9	7	7	7	9	7	7	7	7	7	0	0	12	
20	7	9	7	7	7	9	7	7	7	7	9	9	0	0	12	
21	7	7	7	7	7	7	7	7	7	7	7	7	0	0	12	
22	7	7	9	9	7	7	7	9	7	7	7	7	0	0	12	
23	7	7	9	9	7	7	7	7	9	9	7	9	0	0	12	
24	9	9	7	7	7	9	7	7	7	7	7	7	0	0	12	
F	0	0	0	0	0	0	0	3	0	0	1	0				
B	2	4	1	3	1	0	0	0	4	3	2	0				
P	22	20	23	21	23	24	24	21	20	21	21	24				
Difficulty: b/(b + f)*p/(p + b)	0.917	0.833	0.958	0.875	0.958				0.833	0.875	0.609					

Arrows indicate if the mark is to be modified up or down based on the calculation of Ability and Disability score

* meand multiplication

A simulation study using one of the OBM versions [41] demonstrated that on average the accuracy of the pass/fail decisions made by the OBM was about 70% which is equivalent to effect size = 1 [51]. A more recent study [43], which used real data and applied the OBM2 (the OBM version presented here), yielded 77% accuracy which is equivalent to effect size = 1.4 [51].

This study sought to identify the impact of the implementation of the OBM2 on examination results in a high stakes OSCE early in the Medicine Program, and further, to assess the validity and defensibility of the application of OBM2 to a high stakes OSCE in a Medicine program.

The study was approved by the UNSW Human Research Ethics Advisory (HREA) Panel ref.: HC15421.

## Methods

### Data

This study used data of Phase 1 final OSCE examination (end of Year 2) in the medicine program (*N* = 271).

All original grades generated in the OSCE were modified to the respective ‘raw’ scores (F = 3; B = 5, *P* = 7, P + =9 or 10 if all grades in the station were P+).

The OBM2 was applied to the ‘raw’ scores such that the B grades (score = 5) were modified to either F(=3) or P(=7).

### Statistical analysis

Descriptive statistics were employed to report the distribution of the grades before and after the reclassification. Mean scores and 95%CI were calculated per station and per domain for the unmodified and the modified scores/grades (results are presented on error bars). Similar analysis took place for the P and the F grades which resulted from the modification of the B grades (results are presented on error bars). For comparison of success rate, the OSCE grades were calculated twice. First time using the ‘traditional method’ [45] and then using the OBM2.

Anecdotal feedback from students, examiners and other staff engaged in the OSCE’s was received through committees’ discussions. Quotes could not be provided since these were not covered by the ethics approval but summaries of feedback were added to the discussion as complementary contextual information.

## Results

The OSCE yielded 14,634 grades of which 687 (4.69%) were Borderline (B grade) (Table 2). The application of the OBM2 reclassified 355 (51.7%) of the Borderline grades to Fail and 332 (48.3%) to Pass.

**Table 2:** Grade distribution before and after the implementation of the OBM2

Grade	Score	Prior re-classification		Post re-classification	
		N	%	N	%
F	3	83	0.57	438	3.00
B	5	687	4.69		
P	7	10,338	70.64	10,670	72.91
P+	9	3211	21.94	3211	21.94
P++*	10	315	2.15	315	2.15
Total		14,634	100.00	14,634	100.00

P++ when all grades in a station are P+ Score = 10 (as defined by the program assessment guideline)

**Fig. 1:**
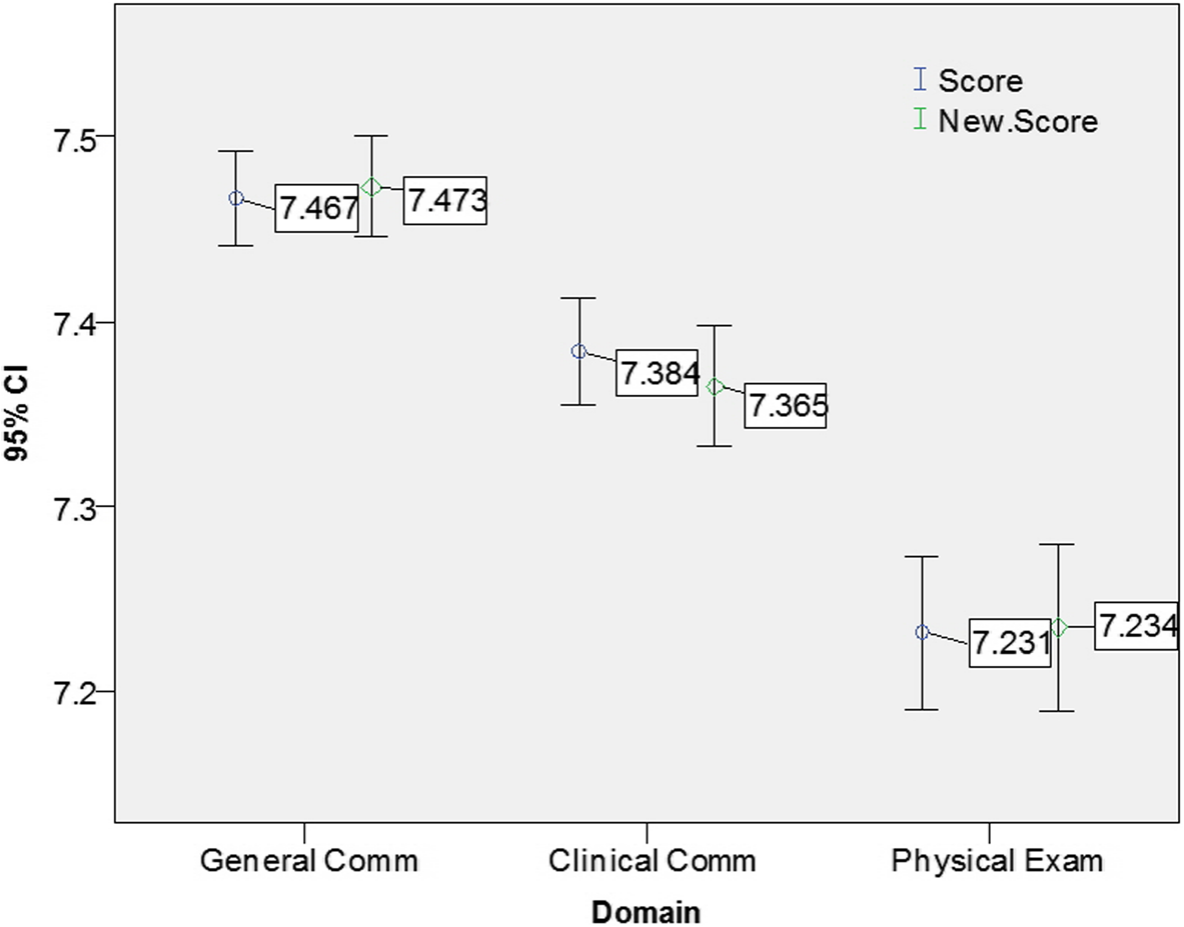
Mean score by domains prior and post reclassification

**Fig. 2:**
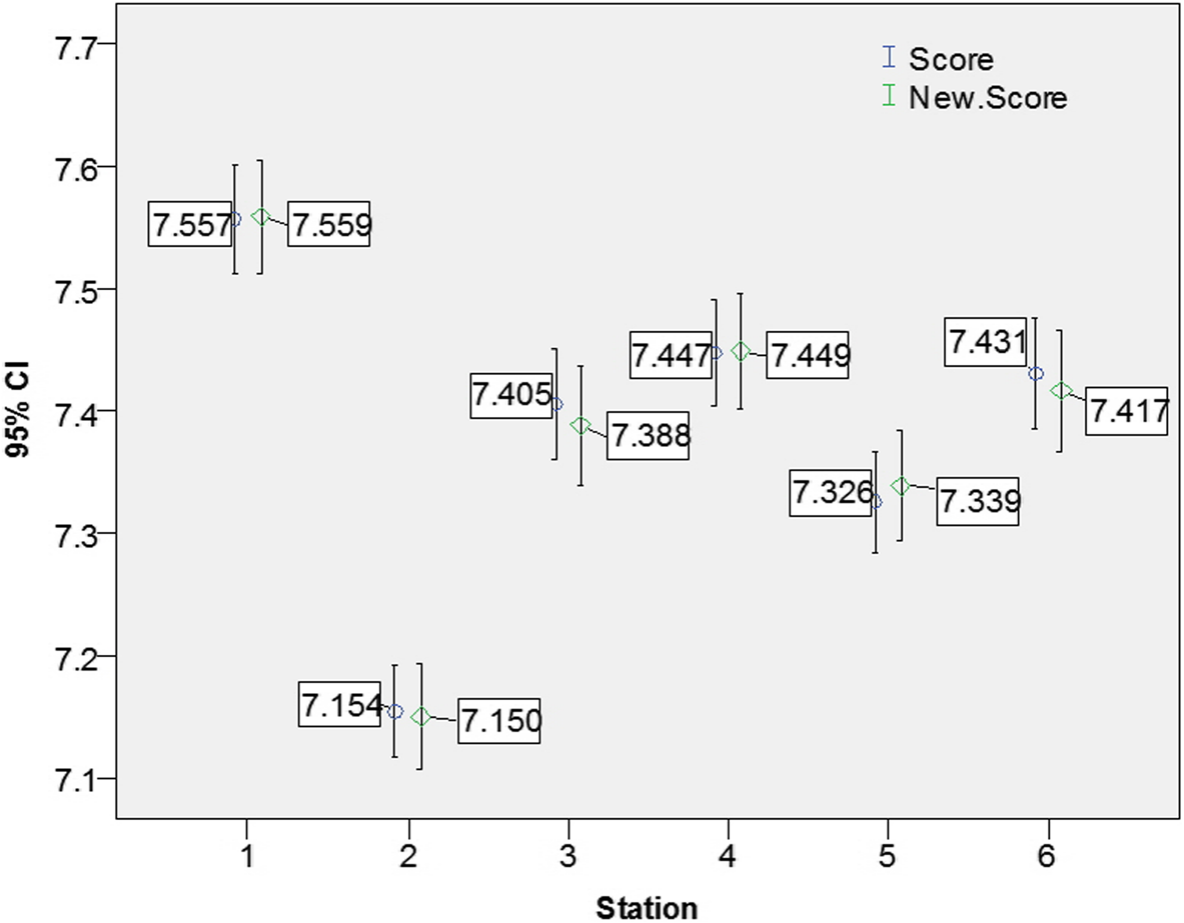
Mean score by station prior and post reclassification

**Fig. 3:**
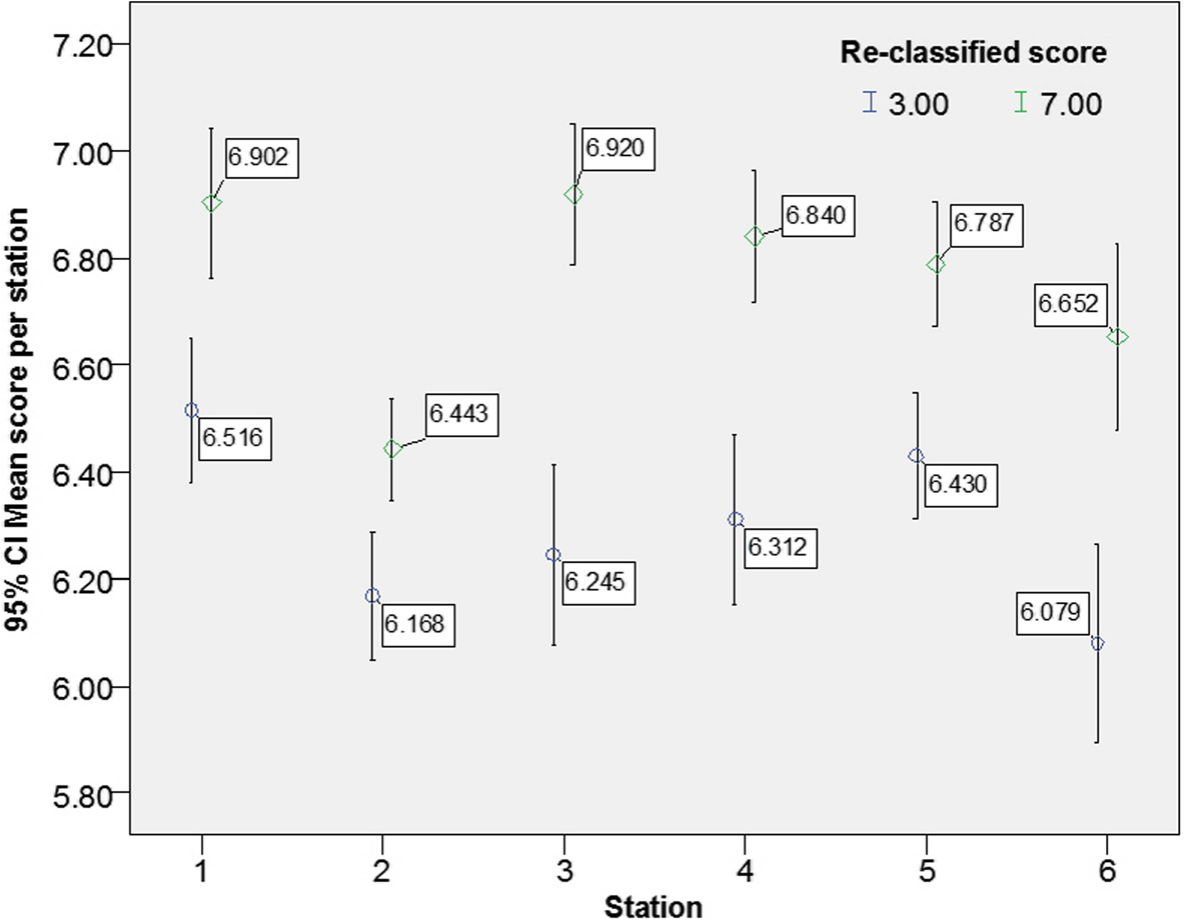
Mean scores of reclassified grades by stations

**Fig. 4:**
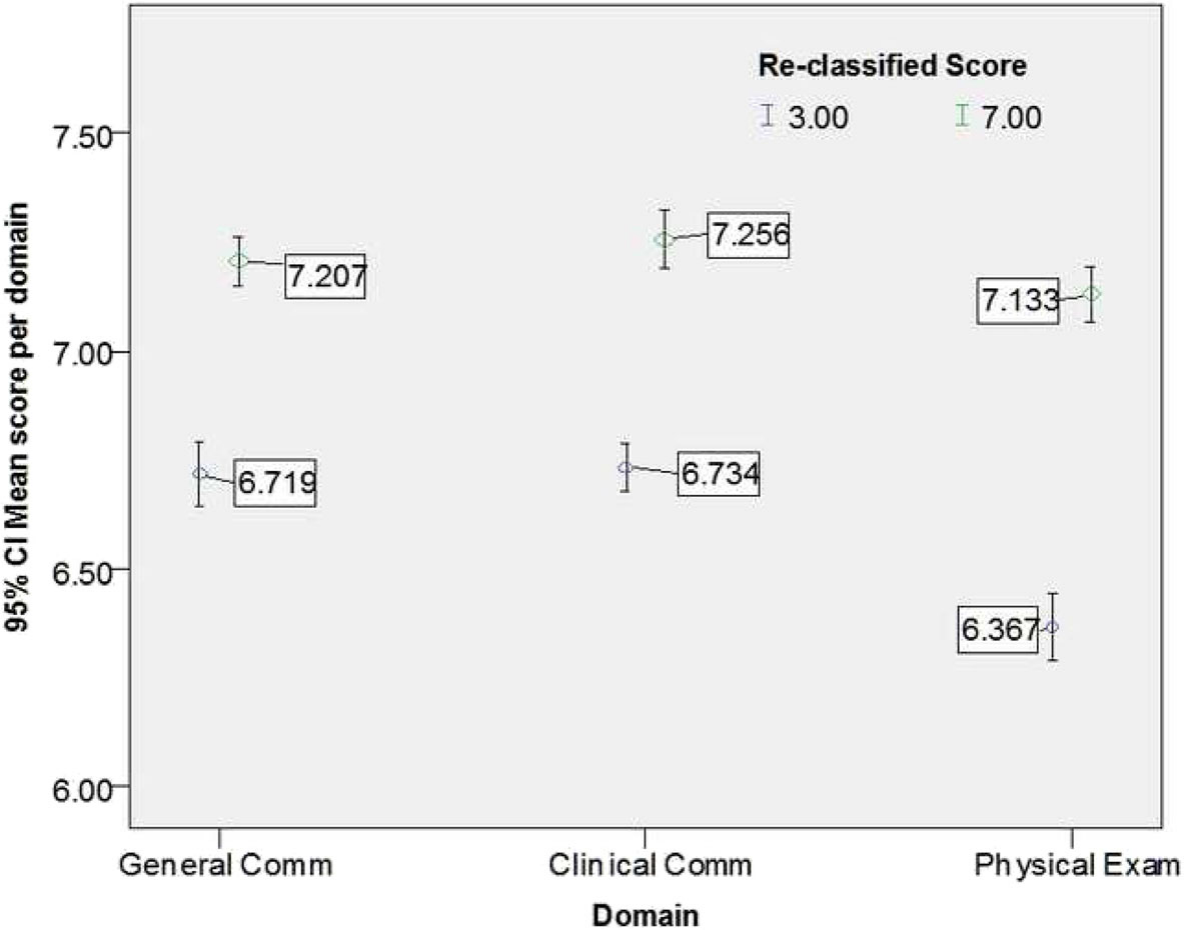
Mean scores of reclassified grades by domains

Passing the OSCE required achieving a mean score ≥ 5 in all three domains and all six stations. The implementation of the OBM2 increased the number of students who failed the OSCE from 2 (0.7%) using the ‘traditional method’ to 11 (4.1%). Figures 1 and 2 demonstrate that the reclassification did not have any significant impact on the mean scores across domains and separately across stations. Nonetheless, Figs. 3 and 4 demonstrate that the mean scores (by stations and by domains respectively) of students whose B grades were modified to P were significantly higher than their counterparts whose B grades were modified to F.

## Discussion

The results of this study clearly demonstrate that the application of the OBM2 to the Phase 1 OSCE at UNSW did impact student outcomes, but in a desirable direction. The most important finding is that the implementation of the OBM2 increased the failure rate from 2 (0.7%) to 11 (4.1%). This major change, more than fivefold, was more reflective of failure rates in other barrier examinations in the Medicine program. Nonetheless, in comparison to the literature a failure rate of 4.1% is within the *lower range* previously reported in clinical examinations [29, 52, 53]. Consequently this outcome (the increase in the failure rate) is perceived as a positive outcome as it increases the confidence that fewer incompetent students passed the OSCE [23, 54, 55] without any expression of dissatisfaction from any stakeholders, particularly students. Moreover, the high level of satisfaction expressed by students, examiners and program leaders throughout the implementation of the OBM2 adds credibility and support to the acceptability of the OBM2 as a method which properly resolves the indecisive borderline grades.

The findings from this study demonstrate that the reclassification made by the OBM2 was appropriate. Figures 3 and 4 demonstrate that B grades that were reclassified to P were associated with higher overall performance whereas reclassification of B to F grades was associated with a lower level of overall performance either across domains within each station or across stations within each domain.

The OBM2 provides a feasible and defensible solution to a relatively overlooked problem: *how to properly assess an examinee’s performance which could not be clearly identified as either pass or fail*. All known standard setting methods aim to establish a cut-score which determines whether the examinees passed or failed the examination (or crossed the boundary for other classifications e.g. pass-distinction). These methods assume that a borderline grade describes a particular level of performance, either just pass (borderline-pass) or just fail (borderline-fail) or in the middle between pass and fail i.e. borderline [38, 56, 57]. In other words the assumption is that a borderline grade describes a discrete level of performance. Logically, this assumption is problematic. One either meets performance criteria or not; it is impossible to both meet and not meet the performance criteria at the same time. Similarly, it is impossible to neither meet nor fail to meet the performance criteria at the same time. Thus, borderline-pass means meeting the performance criteria yet at a lower level and borderline fail means failing to meet the performance criteria but only just. A borderline grade could therefore indicate that there is insufficient information to determine whether the examinee met or did not meet the performance criteria. OBM2 provides a simple yet defensible method for making this determination. When the information is insufficient to determine the examinee’s level of performance, the OBM2 utilises all the ‘real’ assessment interactions between assessors and examinees, to objectively (i.e. without any additional judgement, based on predetermined criteria and using a large number of examiners) determine the most likely level of performance, i.e. pass or fail [2].

An important feature of the OBM2 is that it does not create new performance standards nor does it set any cut-scores. The OBM2 utilises the very same predetermined performance criteria as advised to students, teachers and examiners prior to the examination taking place. This adds fairness since no examinee could be negatively impacted by the OBM2 once the performance criteria have been clearly met; nor would one unjustifiably be granted a pass once the performance criteria have clearly not been met. The OMB2 applies only when the examiner cannot clearly determine the level of the examinee’s observed performance.

An additional advantage of the OBM2 is that it considers the difficulty of each item (i.e. performance criterion). That means that when the OBM2 is applied, the pass/fail decision is more stringent when it is made for an easy item and more lenient when made for a difficult item. This provides an additional layer of fairness to the pass/fail determinations made by the OBM2, and this was noted in student feedback throughout the implementation of the OBM2.

In earlier studies, the OBM and OBM2 were successfully applied to either simulated data or historical data (that is, data that had been generated with no prior intention to apply such methodology) [40–43]. A recent study using OSCE data generated within the OBM framework (borderline = indecisive mark), demonstrated high efficacy of the OBM2 when applied to a common OSCE setting where the pass/fail decisions were made based on assessment marks within a station [50]. The current study demonstrates that the OBM2 is applicable even when pass/fail decisions need to be made based on assessment marks across stations.

Nonetheless, despite the consistent positive outcome yielded from the ‘live’ implementation of the OBM2 in the UNSW Medicine program, there remains a need for further research to better clarify the OBM and OBM2 limitations, particularly but not limited to assessment data that include very small number of students.

## Conclusions

In conclusion, this study demonstrated that the OBM2 is a simple, feasible and defensible method to reclassify indecisive grade (i.e. borderline) to either pass or fail. The OBM2 was found acceptable by all stakeholders and is now fully implemented in a well-established undergraduate Medicine program in Australia. Future studies may provide better insight into the OBM2 including its limitations and advantages.

## Abbreviations

OBM: Objective Borderline Method
OSCE: Objective Structured Clinical Examination
